# The self-assessment scale of cognitive complaints in Schizophrenia: validation of the Arabic version among a sample of lebanese patients

**DOI:** 10.1186/s12888-023-04925-3

**Published:** 2023-06-09

**Authors:** Chadia Haddad, Hala Sacre, Elie Abboche, Pascale Salameh, Benjamin Calvet

**Affiliations:** 1 EpiMaCT - Epidémiologie des maladies chroniques en zone tropicale, Institut d’Epidémiologie et de Neurologie Tropicale, Inserm U1094, IRD UMR270, Univ. Limoges, CHU Limoges, OmegaHealth, Limoges, France; 2 Institut National de Santé Publique, d’Épidémiologie Clinique et de Toxicologie-Liban (INSPECT-LB), Beirut, Lebanon; 3 School of Medicine, Lebanese American University, Byblos, Lebanon; 4 Research Department, Psychiatric Hospital of the Cross, Jal Eddib, Lebanon; 5 School of Health Sciences, Modern University for Business and Science, Beirut, Lebanon; 6 Faculty of Pharmacy, Lebanese University, Hadat, Lebanon; 7 Department of Primary Care and Population Health, University of Nicosia Medical School, Egkomi, Nicosia 2417 Cyprus; 8 Pôle Universitaire de Psychiatrie de l’Adulte et de la Personne Âgée, Centre Hospitalier Esquirol, Limoges, 87000 France; 9 Unité de Recherche et d’Innovation, Centre Hospitalier Esquirol, Limoges, 87000 France

**Keywords:** Schizophrenia, Cognition, Subjective cognitive complaint, Self-Assessment Scale of Cognitive Complaints in Schizophrenia

## Abstract

**Background:**

Several subjective scales have been used to measure cognitive complaints in patients with schizophrenia, such as the Self-Assessment Scale of Cognitive Complaints in Schizophrenia (SASCCS), which was designed to be clear, simple, and easy to use. This study aimed to examine the ability of SASCCS as a validated tool to collect and assess subjective cognitive complaints of patients with schizophrenia.

**Methods:**

A cross-sectional study among 120 patients with schizophrenia was performed between July 2019 and Mars 2020 at the Psychiatric Hospital of the Cross, Lebanon. The SASCCS was used to assess how patients with schizophrenia perceived their cognitive impairment.

**Results:**

The internal consistency of the SASCCS scale was 0.911, and the intra-class correlation coefficient was 0.81 (p < 0.001), suggesting a good stability over time. The factor analysis of the SASCCS scale showed a 5-factor solution using the Varimax rotated matrix. The SASCCS total score positively correlated with their own factors. A negative correlation was found between the objective cognitive scale and subjective cognitive complaints, which were positively correlated with clinical symptoms and depression. No significant association was found between insight and subjective cognitive complaints.

**Conclusion:**

The SASCCS scale showed appropriate psychometric properties, with high internal consistency, good construct validity, and adequate concurrent validity, which makes it valuable for the evaluation of subjective cognitive complaints in patients with schizophrenia.

**Supplementary Information:**

The online version contains supplementary material available at 10.1186/s12888-023-04925-3.

## Background

The importance of cognitive impairment in schizophrenia has recently gained more attention, despite decades of research highlighting both positive and negative symptoms [1–4]. There are different forms of cognitive abnormalities that can vary in intensity from moderate to severe [5] and follow a consistent pattern as the disease advances; they can affect memory, attention, executive functioning, verbal fluency, social cognition, and processing speed [6–8]. Cognitive disability has been shown to affect daily functioning more than positive and negative symptoms and is believed to be the best indicator of patient functional status [1].

Studies have shown that people with schizophrenia often have a dependent personal and functional status and require help and support in various areas, such as problem-solving, basic living skills, and interpersonal and social interactions [9–13]. Consequently, cognitive impairment is now acknowledged as a critical clinical component of schizophrenia [14]. It often occurs before the onset of the core psychotic symptoms and is considered in the evaluation of psychotic disorders in the fifth edition of the Diagnostic and Statistical Manual of Mental Disorders (DSM-5) [15].

Neuropsychological testing can objectively identify cognitive abnormalities, and patients can report their subjective cognitive complaints (SCC) using specific validated scales that reflect their perception of their cognitive functioning [16]. However, some studies have demonstrated weak [17–20] or no association [18, 21] between SCC and objective cognitive impairment, while others have revealed a significant relationship between specific symptoms of schizophrenia and SCC, suggesting that cognitive complaints in schizophrenia may be more a manifestation of a diffuse illness than a patient awareness of cognitive deficits.

Furthermore, new evidence has consistently shown that SCC are more likely connected to mental factors, such as depression and anxiety, than to psychiatric symptoms or cognitive impairment in schizophrenia [22–24]. Overall, a number of variables, including insight, psychotic symptoms, depression, and adverse drug reactions, have been linked to SCC [18, 25, 26]. Hence, it is essential to correctly evaluate the level of insight in schizophrenia patients, especially in light of any cognitive issues. Recent studies have revealed that a significant proportion of patients with schizophrenia had severe cognitive impairments but did not report subjective deficits or display a full awareness of their cognitive disturbances [27, 28]. For this reason, most clinicians rely on neuropsychological assessments to determine the cognitive status of patients rather than self-report measures and often underestimate the degree of cognitive impairment in patients with schizophrenia and their ability to evaluate their cognitive deficits [17].

Several studies have shown that patients with schizophrenia can estimate their cognitive impairment, regardless of their level of insight [17] [18, 29–32], and that they might be aware of their cognitive deficits despite having no insight into their primary condition or other psychiatric symptoms [24, 30, 33, 34]. Also, some evidence shows that SCC are becoming more widely acknowledged as precursors of potential cognitive impairment [35, 36].

It is crucial to give careful consideration to both patient perceptions of their cognitive status and objective tests [37]. While subjective assessments of cognitive functions may offer a more comprehensive understanding of a person’s cognitive profile, they cannot entirely replace objective assessment methods since self-reported neurocognitive functioning might not always be reliable. Nevertheless, subjective evaluation enables patients to become more aware of their symptoms, as it is the first step in the process of psychoeducation and insight building [30]. It is also a valuable tool in the designing individual treatments [38], thereby improving therapeutic relationships and patient willingness to receive care.

Cognitive complaints in individuals with schizophrenia have been measured using various subjective scales such as the Bonn Scale for the Assessment of Basic Symptoms [39], the Frankfurt Complaint Scale [40], the Subjective Experience of Deficits in Schizophrenia [41], the subjective experience interview [42], the Subjective Deficit Syndrome Scale [43], and the Eppendorf Schizophrenia Inventory [44]. These tools address different perceived symptoms of schizophrenia, including subjective cognitive impairment, but do not concentrate on this specific issue. Consequently, two measures have been developed to assess cognitive dysfunctions, the Subjective Scale To Investigate Cognition in Schizophrenia (SSTICS), which is the most widely used tool for assessing self-perception of cognitive function [45–47], and the Self-Assessment Scale of Cognitive Complaints in Schizophrenia (SASCCS) [25]. The latter, created and validated in the Tunisian Arabic dialectic language, showed good internal consistency (Cronbach alpha = 0.85) and an intra-class correlation coefficient of 0.77. This 21-item scale addresses the five cognitive domains most frequently reported in the literature to be impaired in schizophrenia, i.e., memory, attention, executive functions, language, and praxis. It was designed to be clear, simple, and easy to use by patients with schizophrenia [25].

To the best of our knowledge, no scale measuring subjective cognitive complaints has been validated in Lebanon. The authors had previously explored the factors related to SCC among patients with schizophrenia in Lebanon. Therefore, this study aimed to examine the ability of the SASCCS as a validated tool to collect and assess SCC of patients with schizophrenia as part of a goal-directed strategy towards fully understanding subjective cognitive complaints and helping patients with schizophrenia remediate impairments in everyday functioning.

## Methods

### Study design and participants

A cross-sectional study involving 120 long-stay patients with schizophrenia was conducted between July 2019 and March 2020 at the Psychiatric Hospital of the Cross-Lebanon (HPC). Patients had to meet specific criteria to be included, i.e., being between 18 and 60, having at least five years of education, meeting the DSM-5 criteria for schizophrenia, being in a remission phase, taking antipsychotic medication, and being clinically stable. Exclusion criteria included any conditions that could impair cognitive function, such as brain injuries, neurological problems, or ongoing substance use. This study is part of a broader project and used the same methodology as a previous study [17].

### Procedure

The sample was selected from a list generated by the software of the hospital. Out of the 180 individuals who were eligible, 120 were admitted to the study, and 60 were rejected. Of the omitted participants, four had cognitive issues and were unable to finish the evaluation, 22 refused to participate, 21 left the hospital, and 13 declined to proceed with the examination. None of the participants received any payment for their involvement in the study. Well-trained, study-independent professionals collected the data through interviews with the patients.

### Translation procedure

The SASCCS was already available in the Tunisian Arabic dialectic language [25], which differs from the classical Arabic language; therefore, the forward and backward translation method was employed to translate the questionnaire from English into classical Arabic. The forward translation was performed by a mental health specialist, while the backward translation was conducted by another specialist. Subsequently, the two English versions were compared to determine any discrepancies, which were resolved by consensus between the authors. The translated instrument was then pre-tested on two patients with schizophrenia to evaluate their understanding of the questions. No significant issues were found during the pre-testing phase; thus, the translated version was used in the study.

### Measures

The first section of the questionnaire assessed the sociodemographic and clinical characteristics of the participants, including age, gender, educational level, marital status, monthly income, types of schizophrenia, family history of mental disorders, length of hospitalization, duration of illness, and the number of hospitalizations.

The second section of the questionnaire included the following measurements:

#### The self-assessment scale of cognitive complaints in schizophrenia (SASCCS)

The SASCCS is a 21-item self-report instrument that assesses how patients with schizophrenia perceive their cognitive impairment [48]. It includes questions on memory (6 questions: 1–3 and 9–11), attention (5 questions, 12–16), executive functions (3 questions, 17–19), language (2 questions, 20–21), and praxia (5 questions, 4–8) [48]. Items are evaluated on a 5-point Likert scale from 0 (never) to 4 (very often). The SASCCS overall score is determined by summing all the responses [48]. Higher scores indicate more complaints of cognitive impairment. The Cronbach’s alpha value was 0.911.

#### The brief assessment of cognition in schizophrenia (BACS)

The BACS, which is validated in Arabic [49], is a neuropsychological battery that assesses cognitive performance in patients with schizophrenia [50]. It comprises six subscales, i.e., list learning for verbal memory, digit sequencing for working memory, the token motor task for psychomotor function, semantic fluency for verbal fluency, symbol coding for attention and processing speed, and Tower of London for executive function [50]. The Cronbach’s alpha value was 0.853.

#### Assessment of clinical symptoms

The Positive and Negative Syndrome Scale (PANSS) and the Calgary Depression Scale for Schizophrenia (CDSS) were used to evaluate the clinical symptoms.

The PANSS, which has been validated in Arabic [51], is a 30-item questionnaire with three subscales: positive symptoms (7 items), negative symptoms (7 items), and overall psychopathology (16 items) [52]. Each item is given a value between 1 (no symptoms) and 7 (extremely severe symptoms). The total score is calculated by summing the responses, with higher scores indicating more severe symptoms [52]. The Cronbach’s alpha values were as follows: 0.684 (total score), 0.769 (positive symptoms), 0.778 (negative symptoms), and 0.836 (general psychopathology).

The CDSS is a 9-item structured interview measure used to evaluate depression in patients with schizophrenia. It consists of eight structured questions that measure depression, hopelessness, self-depreciation, guilty ideas of reference, pathological guilt, morning depression, early wakening, and suicide, followed by one observational question (observed depression). Higher scores indicated a more severe depression [53]. The Cronbach’s alpha value was 0.839.

#### Insight scale for psychosis (IS)

This self-report survey evaluates the insight levels of individuals with psychotic illnesses [54]. It consists of eight questions with mean scores ranging from 0 to 4 and is divided into three subscales (awareness of illness, re-labeling of symptoms, and need for treatment). The total score is determined by summing the subscale values and ranges from 0 to 12. The higher the score, the greater the insight; the Cronbach’s alpha value was 0.503.

### Data analysis

Data analysis was done using the Statistical Package for the Social Sciences (SPSS) software version 25. A descriptive analysis was performed where categorical variables were expressed as absolute frequencies and percentages and quantitative variables as means and standard deviations. Cronbach’s alpha was calculated for the SASCCS scale to assess its internal consistency reliability. Also, the intraclass correlation coefficient (ICC) was used to test the reliability measure of agreement between two dates. The period between the two measures was long (over 20 months). However, as the test was measuring cognitive function, it was consistent across time; therefore, the scores obtained between these two periods were also consistent [55].

An Exploratory Factor Analysis (EFA) was conducted to identify the factor structure using principal components analysis with Varimax rotation. The Kaiser-Meyer-Olkin measure of sampling adequacy and Bartlett’s test of sphericity were ensured to be adequate. The retained number of factors corresponded to Eigenvalues higher than one. Relationships between SASCCS factors and total scores and clinical variables have been investigated using bivariate correlations. The significance level was set at p < 0.05.

## Results

Table 1 summarizes the sociodemographic characteristics of the participants. The mean SASCCS total score was 25.15 (SD = 16.67; min = 0; max = 76; median = 23.5).

**Table 1:** Sociodemographic characteristics of the studied sample (N = 120)

	Frequency (%)	
Gender		
Male	71 (59.2%)	
Female	49 (40.8%)	
**Education level**		
Complementary	41 (34.2%)	
Secondary	60 (50.0%)	
University	19 (15.8%)	
**Marital Status**		
Single	98 (81.7%)	
Married	10 (8.3%)	
Divorced	10 (8.3%)	
Widowed	2 (1.7%)	
**Monthly income**		
No income	27 (22.5%)	
< 1000 $	64 (53.3%)	
1000–2000 $	27 (22.5%)	
> 2000 $	2 (1.7%)	
**Family history of psychiatric illness**		
Yes	42 (35.3%)	
No	77 (64.7%)	
	**Mean ± SD**	
**Length of hospitalization in years**	12.47 ± 8.56	
**Length of illness in years**	20.64 ± 9.79	
**Number of hospitalizations**	6.32 ± 5.65	
**Age in years**	48.43 ± 7.62	

### Reliability

#### Internal consistency

The internal consistency measured by Cronbach’s alpha coefficient was 0.911.

#### Test-retest reliability

The test-retest was assessed within a subgroup of 95 patients examined by another investigator at a mean interval of 20.65 ± 1.47 months. The intra-class correlation coefficient was equal to 0.81 (95% Confidence Interval: 0.71–0.87; p < 0.001), suggesting good stability over time.

#### Validity of internal structure

A factor analysis was run to test the construct validity of the SASCCS scale using the principal component analysis as the extraction method. All items of the SASCCS scale could be extracted from the list, and the scale converged on a 5-factor solution using the Varimax rotated matrix with an eigenvalue greater than 1, accounting for 65.19% of the variance (Bartlett sphericity test p < 0.001, KMO = 0.870) (Table 2). In addition, the factor analysis was done by using the maximum likelihood method with the Promax rotation method, and approximately the same factors were found (Supplementary Table 1). When comparing the two methods of factor analysis, the results showed that Factors 1, 3, and 4 in the initial factor analysis were almost the same as the second analysis. There were differences in Factor 2 (verbal memory) and Factor 5 (disorder consciousness), where some items were mixed between these two factors showing that memory is related to different types of consciousness [56].

**Table 2:** Factor analysis. Rotation Varimax

	Items	Factor 1	Factor 2	Factor 3	Factor 4	Factor 5
Do you have difficulties to organize your daily activities? Such as shopping, cooking, cleaning the house, fixing stuff, doing some laundry	18	0.908				
Do you have difficulties planning something in advance? Example, updating your health care card, getting some money from your post office account, or planning how to spend your budget for the month?	17	0.793				
Do you have difficulties to do usual activities? Example, to dress up or button a shirt, to introduce a key in a lock, to use a spoon?	21	0.682				
Do you have difficulties to change your way of thinking or your manner of doing something the way you’re used to do it when you’re asked to do so and you agree to make these changes?	19	0.584				
Do you have difficulties to focus on something for more than 20 min? Example, listening to the news, reading a magazine, watching a sitcom, attending a school lesson	16	0.570				
Do you feel like you have memory disturbances?	1		0.837			
Do you have difficulties to retain something in your mind? Example, a shopping list or a list of persons’ names	3		0.815			
Do you have any problems to remember information you’ve just learned and that you should immediately use? Example, an address, a telephone number, a bus number, a doctor’s name	2		0.782			
Do you have any problems to remember information you learned in a paper or watched on TV yesterday?	7		0.523			
Have you ever forgotten how to cook a dish or which ingredients you should put in / Have you ever forgotten how to fix or repair things at home	8		0.508			
Do you have any problems remembering names of people belonging to fields you’re usually interested in? (sports, cinema, songs…)	10		0.436			
Do you have any difficulties staying in alert and reacting quickly when something you didn’t expect happens? Example, avoiding a car when crossing the street	13			0.772		
Do you have any problems remembering names of the biggest towns in Tunisia or the most important historical events of your country, or the names of the biggest cities in the world?	11			0.693		
Do you have difficulties to find your words, to make sentences, to understand the meaning of some words, to pronounce them, to designate objects by their name	20			0.646		
Do you feel like you are distracted for example when speaking with someone or reading a magazine?	12			0.620		
Have you ever forgotten an appointment with your friend or with your doctor?	5			0.598		
Do you have any problems to find your way by yourself to the hospital, the outpatient clinic or even to your home?	9			0.527		
When the television is on and people around are talking loudly, do you have any difficulties to focus on a particular conversation?	14				0.816	
Do you have difficulties to do 2 different things at the same time? Example, having a conversation with someone while watching television, or doing some housekeeping while cooking a lunch on the gas stove	15				0.808	
Do you sometimes forget to take your treatments?	6					0.859
Do you have any problems to remember the name of your treatments?	4					0.474
Percentage of variance explained = 65.19%		36.80	10.33	7.77	5.51	4.77
KMO: 0.870 ; Bartlett’s Test of Sphericity < 0.001						

Factor 1: organizing daily activity ; Factor 2: Verbal memory; Factor 3: Distractibility; Factor 4: Executive skills; Factor 5: Disorder consciousness

### Convergent validity

The SASCCS total score positively correlated with its own factors. Also, a positive correlation was found between the factors (Table 3).

**Table 3:** Correlation analysis between the factors and the total SASCCS scale

	Factor 1	Factor 2	Factor 3	Factor 4	Factor 5
**Total scale**	0.763***	0.873***	0.545***	0.564***	0.594***
Factor 1	-	0.507***	0.509***	0.279**	0.302**
Factor 2		-	0.360***	0.477***	0.488***
Factor 3			-	0.258*	0.288**
Factor 4				-	0.286**
Factor 5					-

Note: p-value ***<0.001; **<0.01; *<0.05

**Factor 1: organizing daily activity; Factor 2: Verbal memory; Factor 3: Distractibility; Factor 4: Executive skills; Factor 5: Disorder consciousness**

The correlation between SASCCS total scale and cognition (BACS), clinical symptoms (PANSS and CDSS scales), and the insight scale are reported in Table 4. A negative correlation was found between the total cognitive scale, subscales, and subjective cognitive complaints (higher complaints – less severe cognitive function), except for the attention and speed of information processing, where no significant association was found. A positive correlation was found between PANSS total score and subscales, depression, and subjective cognitive complaints (higher complaints – more severe depression and symptoms). No significant association was found between insight and subjective cognitive complaints.

**Table 4:** Correlations between the SASCCS scale and quantitative scales

	SASCCS total score	p-value
BACS total score		
Verbal memory	-0.425	**< 0.001**
Working memory	-0.321	**< 0.001**
Motor speed	-0.314	**< 0.001**
Verbal fluency	-0.334	**< 0.001**
Attention and speed of information processing	-0.077	0.403
Executive function	-0.211	**0.021**
**PANSS total score**	0.394	**< 0.001**
Positive PANSS scale	0.227	**0.013**
Negative PANSS scale	0.183	**0.045**
General psychopathology PANSS scale	0.420	**< 0.001**
**Insight Scale for psychosis**	0.077	0.401
**Depression scale**	0.331	**< 0.001**

## Discussion

In this study, the SASCCS was translated into the classical Arabic language and validated among a sample of Lebanese patients with schizophrenia. The validated version demonstrated strong internal consistency, stability over time, and good construct and convergent validity, showing that the tool is valuable for the assessment of self-perceived cognitive impairment in patients with schizophrenia. A high internal consistency was found (Cronbach’s alpha = 0.91), comparable to other findings [25, 57], suggesting that SASCCS is a reliable measure for cognitive complaints.

Based on EFA results, the factor analysis found a 5-factor structure that differed from the original version in which the scale was built [25]. In the original study, using EFA, the authors proposed a 6-factor structure, accounting for a cumulative 58.28% of the variance [25]; our 5-factor construct accounts for a slightly higher percentage of variance explained (65.19% of the total variance). In the Spanish version, the SASCCS factor analysis produced two scales with 5 and 6 factors, with the last factor being the one that better adjusts [57]. A hidden factor was retained after the Oblimin rotation, accounting for the 6-factor model, with a slightly higher proportion of variance explained (variance explained was 55.25% for the five factors and 60.26% for the six factors). Overall, the 5-factor structure found in the current study confirmed the original conceptual model of five domains (attention, memory, activity of daily life, executive function, and disorder consciousness), despite the disparity captured in the memory domain in the original article [25]. Additionally, the specificity of the items might be affected since many cognitive factors overlap. Therefore, these findings support the complex representation that people with schizophrenia have of their own cognition, which differs from one population to another and does not match exactly their theoretical constructs [58].

The SASCCS scale was negatively correlated with the BACS total scale and subscales, indicating a reverse association between subjective and objective measures of cognition. Our findings are similar to those of previous studies [29, 30, 57] but contradictory with others showing no correlation between objective and subjective cognitive function [18, 59, 60]. A possible explanation would be that patients complain more frequently about their cognitive functioning than their objective cognitive deficits, suggesting that they might be aware of these deficits and able to express their cognitive functioning subjectively. Another possible explanation is that the subjective complaints of cognitive problems coincided with objective performances in those same dimensions. There was also a positive correlation between the cognitive PANSS subscale and the SASCCS scale, which again suggests that the subjective assessment of cognition overlaps with the objective assessment of dimensions in patients with schizophrenia. The general psychopathology subscale covers many deficits in cognition, such as disorientation, poor attention, lack of insight, and active social avoidance [61], which could overlap with the subjective cognitive domains. However, in the original study among 105 patients with schizophrenia spectrum disorders, no correlation was found between the SASCCS scores and the PANSS cognitive factor, considering that the subjective assessment of cognition in individuals with schizophrenia may be a separate dimension from its objective assessment [25].

Our study found a significant positive correlation between positive and negative symptoms and cognitive complaints, consistent with previous findings revealing similar associations between SCC and PANSS scores [29, 31]. In contrast, other results showed that these associations were in the reverse direction. A study in Tunisia found that the SASCCS total score was not correlated to the PANSS scale and subscales [25]. Another study among 115 patients with schizophrenia showed a positive association between positive symptoms and cognitive complaints but did not find a correlation between negative symptoms and cognitive complaints [18]. In Italy, a positive association between the negative PANSS scale and SCC was reported among 146 patients with schizophrenia [62], with the most relevant correlation of the negative PANSS items being between PANSS depression and SCC [62]. Of note, schizophrenia patients may assess their social cognitive abilities less accurately, either overestimating or underestimating their performance [63].

Consistent with other studies [25, 31, 62], a positive correlation was found between the SASCCS total score and depression, suggesting that the patient reports cognitive problems more frequently, the more severe the depression symptomatology. One of the reasons for the association between depression and SCC is that those who experience it may be more prone to blame memory loss or cognitive issues for their troubles [64]. Patients with depression could be overly sensitive to commonly occurring cognitive dysfunctions and self-conscious of their cognitive ability.

The present study failed to demonstrate an association between SCC and insight, supporting earlier findings showing that individuals with schizophrenia may be aware of their cognitive deficiencies while having little understanding of their symptoms or disease [24, 30, 33]. Therefore, awareness of cognitive impairments can occur without understanding the symptoms of the disease and independently of insight. However, this association between insight and SCC was found in other studies [18, 25, 62], suggesting that being aware of one’s overall mentally sick status may significantly affect awareness of one’s cognitive deficiencies. Due to the conflicting results found in the literature, more studies are necessary to evaluate patient insight when using the SASCCS.

### Limitations

Our study has several limitations. Its cross-sectional design does not allow consequent validity to be inferred. Since the population consists of chronically hospitalized patients whose cognitive function might be severely impaired, selection bias is possible. Moreover, the study results cannot be generalized to the population due to the small sample size and the fact that patients were selected from one site. Information bias might have occurred because participants were unable to give accurate details in the face-to-face interview. The factor analysis showed that factors with two items were kept; however, these factors might be unstable because they comprise only two items, which might have affected the evaluation of patient insight. Thus, further studies are warranted to confirm our findings.

## Conclusion

The SASCCS scale showed appropriate psychometric properties with high internal consistency, good construct validity, and adequate concurrent validity, making it valuable for assessing subjective cognitive complaints in patients with schizophrenia. Consequently, the SASCCS can be considered an easy-access tool that might be readily included in standard clinical settings and research studies. More research is required to verify our findings and examine other aspects of SASCCS scale reliability, validity, and cut-off factors.

## Electronic supplementary material

Below is the link to the electronic supplementary material.

**Supplementary Table 1**: Factor analysis using the maximum likelihood method with Promax Rotation

## Data Availability

The datasets used and/or analysed during the current study are available from the corresponding author on reasonable request.

## List of abbreviations

SASCCS: Self-Assessment Scale of Cognitive Complaints in Schizophrenia
DSM-5: Diagnostic and Statistical Manual of Mental Disorders, fifth edition
SCC: Subjective cognitive complaints
SSTICS: Subjective Scale To Investigate Cognition in Schizophrenia
HPC: Psychiatric Hospital of the Cross-Lebanon
BACS: Brief Assessment of Cognition in Schizophrenia
PANSS: Positive and Negative Syndrome Scale
CDSS: Calgary Depression Scale for Schizophrenia
IS: Insight Scale for Psychosis
SPSS: Statistical Package for the Social Sciences
EFA: Exploratory Factor Analysis
SD: Standard deviation
KMO: Kaiser-Meyer-Olkin
